# Smoking and γ-Glutamyltransferase: Opposite Interactions with Alcohol Consumption and Body Mass Index

**DOI:** 10.1371/journal.pone.0013116

**Published:** 2010-09-30

**Authors:** Lutz P. Breitling, Volker Arndt, Christoph Drath, Dietrich Rothenbacher, Hermann Brenner

**Affiliations:** 1 Division of Clinical Epidemiology and Aging Research, German Cancer Research Center, Heidelberg, Germany; 2 Workmen's Compensation Board for Construction Workers, Occupational Health Service, Böblingen, Germany; VTT Technical Research Centre of Finland, Finland

## Abstract

**Background:**

Smoking has recently been suggested to synergistically interact with alcohol intake as a determinant of serum gamma-glutamyltransferase (γ-GT), an emergent powerful predictor of disease and mortality. This study investigated whether this also applies to higher smoking and alcohol exposure ranges and to body mass index (BMI), which likewise is strongly associated with γ-GT.

**Methodology/Principal Findings:**

Analyses were based on occupational health examinations of more than 15,000 German male workers aged 16–64 years, predominantly from the construction industry. Sociodemographics and other health-related information were collected during the exam. Joint associations of smoking and alcohol consumption or BMI with elevated or log-transformed γ-GT were examined by tabulation and multiple adjusted regression models. Cigarette smoking exerted no effect on γ-GT in teetotalers, but there was a statistically significant effect of smoking among participants with higher alcohol consumption intensity, odds of elevated γ-GT being increased by 24% and 27% per additional 10 cigarettes smoked per day in subjects drinking 61–90 and >90 gram alcohol per day, respectively (*P* for interaction = 0.039). The interaction was opposite for BMI, where no association was seen in obese subjects, whereas odds of elevated γ-GT were increased by 24% per 10 cigarettes below 25 kg/m^2^ (*P* for interaction = 0.040). This novel interaction was replicable in an independent cohort.

**Conclusion:**

The evidence for opposite interactions of smoking with alcohol and BMI as determinants of serum γ-GT suggests that different physiological pathways are responsible for the associations between these factors.

## Introduction

The liver enzyme gamma-glutamyltransferase (γ-GT) is an emergent risk marker for a variety of common diseases such as diabetes and cancer [1], [2], [3], [4], as well as for overall and cause-specific mortality [5], [6], [7], [8]. Better understanding factors determining individual γ-GT levels thus has become a quest of high relevance to public health. Whereas alcohol consumption certainly remains the single most prominent γ-GT predictor, significant associations with additional modifiable factors such as body mass index (BMI), coffee consumption and smoking have been shown repeatedly [1], [2], [8], [9], [10], [11], [12].

In a recent study conducted in an elderly population-based cohort with relatively moderate smoking prevalence and alcohol consumption, we found no relevant main effect of cigarette smoking on γ-GT levels after adjustment for covariables. However, a detrimental interaction between smoking and alcohol was found, with γ-GT appearing more sensitive to alcohol consumption in smoking than non-smoking subjects [13]. As smoking and alcohol drinking behaviour are strongly correlated, such exacerbating interaction effects—if their existence was confirmed—would apply to a large fraction of the population.

Other data investigating the interaction between smoking and alcohol consumption are scarce [14], [15], and the underlying pathophysiological mechanisms remain unclear. A plausible hypothesis might be that oxidative stress imposed by both smoking and alcohol surpasses some threshold intensity more easily during joint exposure [13], leading to excessive γ-GT release and/or induction. If this was true, a similar interaction would be expected with BMI, which—like alcohol consumption—is positively associated with both oxidative stress [16] and γ-GT [6], [13]. Therefore, we attempted to corroborate our previous findings by extending them to an independent study population featuring especially high smoking and alcohol consumption intensities, and to gain further physiological insights by additionally examining potential interactions between smoking and BMI.

## Methods

### Ethics Statement

Whereas participation in the occupational health exams forming the basis of this work is non-mandatory for most occupation groups according to German occupational safety laws, anonymised data obtained in such exams is to be collected and analysed scientifically. Thus, no additional specific informed consent was required for analysis of anonymised data in this project. However, the study protocol was approved by ethics committees of the Universities of Heidelberg and Ulm, and by the Baden-Württemberg state ministry of social affairs.

### Design, Setting and Participants

The present work was carried out using data obtained as baseline information for a prospective cohort investigation of disability and mortality in construction workers in the south of Germany [6], [8], [17]. In brief, all employees in Germany are entitled and invited to routine occupational health examinations free of charge. Male subjects participating in such an examination (>75% of those invited during the recruitment period) at any of eight health centers of the Workmen's Compensation Board for construction workers in Württemberg from August 1986 to December 1992 were included in this study.

### Data Collection

Occupational health examinations were conducted by experienced occupational health physicians and included detailed standardised questionnaires regarding occupational and life style factors, physical and functional exams, and laboratory measurements. Collected data of relevance to the present analysis included sociodemographics, body weight and height, information on health-related behaviours (smoking, drinking), and prevalent morbidity coded according to the International Classification of Diseases revision 9 (ICD-9).

Based on pertinent self-reports, participants were classified into never, former, or current smokers. Current smokers were categorised by smoking intensity (<20, 20, or >20 cigarettes per day). The amount of pure alcohol consumed per day was estimated during the health exam based on questions referring to common consumption units of alcoholic beverages in the study region [6], and categorised in five groups: no alcohol consumption (teetotalers), 1–30, 31–60, 61–90 and >90 g/day. Subjects for whom ‘occasional’ alcohol consumption had been recorded without further information were treated as a separate category. Serum concentrations of γ-GT were measured centrally as part of the occupational health exam, using a Hitachi 705/717 instrument working at 25°C with an upper reference limit (according to manufacturer specifications) for males of 28 U/L.

### Statistical Analysis

The study population was first characterised regarding sociodemographics (age, nationality, occupational group), main exposures, and covariables potentially associated with both exposures and serum γ-GT. The latter included BMI categorised as <25 (normal weight), 25 to <30 (overweight), and ≥30 kg/m^2^ (obese; [18]), prevalent diabetes (ICD-9 250), ischemic heart disease (ICD-9 410–414), or hypertension (ICD-9 401–405). The distribution of γ-GT over other variables was examined by tabulating geometric means and associated 95% confidence intervals, as well as proportions of subjects with elevated γ-GT (>28 U/L). The statistical significance of associations between the covariables and the latter were examined by logistic regression models controlling for age and BMI. Unless stated otherwise, all exposures were coded as dummy variables. For smoking and alcohol trend models, all participants in each category were assigned the median smoking/consumption intensity of the respective stratum.

Potential interaction effects of joint smoking and alcohol exposure or smoking and BMI category were examined by tabulating γ-GT concentrations over smoking × alcohol consumption and smoking × BMI categories, respectively. The associations then were quantified in logistic regression models predicting elevated γ-GT and linear regression models predicting natural log (*ln*)-transformed γ-GT from each such category in reference to never smoking teetotalers (smoking × alcohol models) or in reference to never smoking subjects with BMI <25 kg/m^2^ (smoking × BMI models). The linear regression models with *ln*(γ-GT) as the outcome avoid the potential pitfalls of dichotomisation [19] and allow the estimation of the relative change in concentrations [20]. Finally, we examined models including an interaction between smoking intensity and alcohol consumption or BMI, and additionally explored the interaction of alcohol consumption with BMI. The significance of the interaction was assessed by a Wald test with 5 and 2 degrees of freedom in the case of the interaction with alcohol consumption and BMI, respectively. Statistical analyses were carried out using SAS 9.2 for Windows (SAS Institute, Cary, NC, USA), tests being two-sided with α = 0.05 throughout.

### Ad hoc Replication Study

Data from the baseline examination of the population-based ESTHER cohort study previously described in detail [13], [21] were used for replication analyses of the smoking × BMI interaction. In brief, elderly women and men (age 50 to 74) presenting for a general health check-up exam at their general practitioner in the German state of Saarland between 2000 and 2002 were included in this study. Information on health behaviours and disease history were obtained from self-administered questionnaires. Serum γ-GT was measured using an assay working at 37°C with an upper reference limit (according to manufacturer specifications) of 50 U/L for males. Differences in the statistical procedures in comparison to the present main analyses were due to differences in the distribution and availability of covariable data and included a different definition of smoking (none, 1–10, 11–20, 21–30, >30 cig./d) and alcohol consumption classes (none, 1–39, 40–99, 100–199, ≥200 gram per week), additional adjustment for sex and coffee consumption, and sex-stratified analyses.

## Results

### Description of the Study Population

For the present study, overall 22,124 individual health screening exam records including data on γ-GT concentrations were available. Of these, 17,749 featured information on smoking, leaving 17,487 after exclusion of pipe and cigar smokers. Due to insufficient information on alcohol consumption, 15,674 (70.8%) of the 22,124 subjects ultimately remained in the final analysis set.

Table 1 provides information on main characteristics of the subjects analysed. The median (interquartile range; IQR) age was 41 (29–51) years. The majority were of German nationality. Almost a third of the cohort were bricklayers. Current smokers mostly reported heavy smoking, with a median (IQR) of 20 (12–20) cigarettes per day. While 11.5% of the participants were classified as teetotalers, self-reported regular alcohol consumption was fairly high in the remaining subjects for whom quantitative data were available (n = 7641), with a median (IQR) of 60 (40–80) grams alcohol per day. The median (IQR) of the BMI distribution equalled 25.8 (23.5–28.4) kg/m^2^. The γ-GT concentration showed a median (IQR) of 17 (12–31) U/L and ranged from 1 to 2252 U/L, with a 99th percentile of 246 U/L.

**Table 1:** Description of study participants.

				Serum γ-GT (U/L)					
Characteristic	Group	n	%	Geom.mean, 95% CI				>28 U/L	*P* a
Age	<25 years	1664	10.6	12.9	12.5	-	13.3	8.9%	ref.
	25-34 years	4434	28.3	18.1	17.7	-	18.6	22.5%	<0.0001
	35-44 years	2984	19.0	23.7	22.9	-	24.6	34.8%	<0.0001
	45-54 years	4430	28.3	23.9	23.2	-	24.5	35.1%	<0.0001
	≥55 years	2162	13.8	22.7	21.9	-	23.5	32.0%	<0.0001
Nationality	German	11894	76.1	21.3	20.9	-	21.7	30.1%	ref.
	Italian	1104	7.1	19.7	18.8	-	20.7	25.3%	<0.0001
	Turkish	839	5.4	13.3	12.7	-	13.8	7.0%	<0.0001
	Yugoslavian	1317	8.4	20.3	19.3	-	21.4	28.3%	<0.0001
	Other	472	3.0	19.2	17.7	-	20.8	26.1%	0.0362
Occupational group	Bricklayer	4801	30.6	21.8	21.2	-	22.4	31.1%	ref.
	Plumber	2380	15.2	19.4	18.7	-	20.1	26.0%	0.058
	Carpenter	2111	13.5	18.9	18.2	-	19.6	24.7%	0.0025
	Painter	2338	14.9	20.9	20.1	-	21.6	28.7%	0.82
	Plasterer	1595	10.2	21.1	20.1	-	22.0	30.2%	0.76
	Unskilled	2242	14.3	20.2	19.5	-	21.0	27.0%	0.0042
	Office	207	1.3	17.6	15.9	-	19.6	19.3%	0.0011
Cigarette smoking	Never	4209	26.9	18.3	17.8	-	18.8	22.7%	ref.
	<20 cig./d	3004	19.2	18.9	18.3	-	19.6	24.9%	<0.0001
	20 cig./d	3798	24.2	20.7	20.1	-	21.2	28.8%	<0.0001
	>20 cig./d	1971	12.6	24.4	23.3	-	25.5	36.0%	<0.0001
	Formerly	2692	17.2	23.4	22.6	-	24.3	34.4%	<0.0001
								Trend^b^	<0.0001
Alcohol consumption	None	1807	11.5	13.6	13.2	-	14.0	8.9%	ref.
	Occasional	6226	39.7	16.5	16.2	-	16.8	18.2%	<0.0001
	1-30 g/d	1613	10.3	18.4	17.7	-	19.1	22.3%	<0.0001
	31-60 g/d	2984	19.0	24.2	23.4	-	25.0	36.2%	<0.0001
	61-90 g/d	1492	9.5	31.6	30.0	-	33.2	49.5%	<0.0001
	>90 g/d	1552	9.9	42.3	40.1	-	44.6	61.8%	<0.0001
								Trend^b^	<0.0001
Body mass index	<25 kg/m^2^	6268	40.8	16.3	15.9	-	16.6	17.8%	ref.
	25-<30 kg/m^2^	6947	45.2	22.6	22.2	-	23.1	32.3%	<0.0001
	≥30 kg/m^2^	2158	14.0	29.5	28.4	-	30.6	46.3%	<0.0001
Prevalent diabetes	no	15000	95.7	20.1	19.8	-	20.4	27.4%	ref.
	yes	674	4.3	32.6	30.1	-	35.3	48.7%	<0.0001
Prevalent IHD^c^	no	15447	98.6	20.5	20.1	-	20.7	28.1%	ref.
	yes	227	1.4	25.9	23.1	-	29.1	37.9%	0.44
Prevalent hypertension	no	12496	79.7	18.6	18.3	-	18.9	23.5%	ref.
	yes	3178	20.3	30.3	29.3	-	31.4	47.1%	<0.0001

a *P*-value from logistic regression predicting γ-GT>28 U/L, adjusted for age and/or BMI.

b Former smokers excluded from smoking trend model (category medians: 0, 10, 20, 30 cig./day), occasional drinkers excluded from alcohol trend model (category medians: 0, 20, 50, 75, 100 g/day).

c IHD  =  Ischemic heart disease.

Levels of serum γ-GT (at 25 °C) in n = 15,674 working age males in Southern Germany, according to sociodemographics, occupation, nationality, life-style factors and prevalent diseases.

### Bivariate Analyses of γ-GT Concentrations

Proportions of subjects with elevated γ-GT and geometric mean concentrations over covariables are also presented in Table 1. Associations with all variables but prevalent ischemic heart disease were statistically significant. The trends were generally consistent with expectations, in particular with increases with higher age and smoking intensity, and with pronounced increases with higher alcohol consumption and BMI. For smoking and alcohol drinking intensities, trend models were fitted and confirmed the presence of a statistically significant trend.

### Interaction Analyses: Tabulations

To examine the potential interaction effect of smoking and alcohol consumption with respect to γ-GT concentrations, we first tabulated crude geometric mean concentrations by smoking × drinking categories (Table 2). Within the alcohol drinking groups, there was a weak tendency for elevated γ-GT concentrations with high smoking intensity, but the patterns were not entirely consistent. For example, never smokers showed higher mean γ-GT than smokers of 20 cigarettes per day in both alcohol-abstinent subjects and those drinking >90 g/day, and former smokers featured the highest mean γ-GT levels in multiple alcohol strata. Note, however, that both never and former smokers featured both higher median age (44 and 48 years) and BMI (26.2 and 27.3 kg/m^2^) than subjects smoking <20, 20, or >20 cigarettes per day (33, 36, 39 years, and 25.0, 25.0, 25.4 kg/m^2^, respectively).

**Table 2:** Geometric mean γ-GT (U/L at 25 °C) according to smoking and alcohol consumption category.

	Alcohol consumption																													
Smoking	None					Occasional					1-30 g/day					31-60 g/day					61-90 g/day					>90 g/day				
behaviour	n	Mean	95% CI			n	Mean	95% CI			n	Mean	95% CI			n	Mean	95% CI			n	Mean	95% CI			n	Mean	95% CI		
Never	752	13.6	12.9	-	14.3	1856	16.1	15.6	-	16.6	449	17.8	16.6	-	19.1	629	23.2	21.6	-	24.8	298	28.6	25.6	-	32.0	225	42.6	36.6	-	49.6
<20 cig./d	267	13.1	12.1	-	14.2	1301	15.2	14.6	-	15.8	356	17.1	15.7	-	18.7	605	23.3	21.7	-	25.1	247	31.3	27.5	-	35.7	228	39.8	34.4	-	46.1
20 cig./d	377	13.0	12.3	-	13.7	1422	16.2	15.6	-	16.8	376	19.1	17.6	-	20.8	769	23.3	21.9	-	24.9	425	31.4	28.6	-	34.5	429	39.6	36.0	-	43.5
>20 cig./d	168	14.2	12.8	-	15.9	628	18.1	17.0	-	19.2	148	18.3	16.4	-	20.5	385	24.8	22.5	-	27.4	252	31.9	28.3	-	35.9	390	45.8	41.2	-	51.0
Formerly	243	14.8	13.6	-	16.0	1019	19.0	18.1	-	19.9	284	20.0	18.2	-	21.9	596	27.1	25.2	-	29.2	270	35.3	31.2	-	40.0	280	43.8	38.7	-	49.5

As shown in Table 3, the pattern emerging in tabulations of mean γ-GT by smoking and BMI category revealed a somewhat different picture. Whereas γ-GT showed a clear positive association with BMI independent of smoking behaviour, an increase in γ-GT with higher smoking intensity was evident in normal weight and suggestive in overweight subjects, but essentially absent in obese subjects. As above, the former smokers appeared to be somewhat outside of the dose-response relationship.

**Table 3:** Geometric mean γ-GT (U/L at 25 °C) according to smoking and body mass index category.

	Body mass index														
Smoking	<25 kg/m^2^					25 to <30 kg/m^2^					≥30 kg/m^2^				
behaviour	n	Mean	95%CI			n	Mean	95%CI			n	Mean	95%CI		
Never	1429	13.6	13.1	-	14.1	2091	20.0	19.3	-	20.8	623	27.0	25.2	-	29.0
<20 cig./d	1466	14.7	14.1	-	15.3	1128	22.9	21.8	-	24.2	346	30.0	27.2	-	33.2
20 cig./d	1862	17.5	16.8	-	18.2	1487	23.2	22.2	-	24.2	371	29.5	27.0	-	32.3
>20 cig./d	874	21.8	20.3	-	23.3	791	25.8	24.2	-	27.6	268	30.4	27.3	-	33.8
Formerly	637	16.9	15.8	-	18.1	1450	24.3	23.2	-	25.5	550	31.5	29.3	-	33.9

### Interaction Analyses: Regression Models

Results of detailed regression analyses regarding the smoking × alcohol interaction are provided in Supplementary Table S1. In the models adjusted only for age and BMI, the pattern of very high γ-GT in never and former smokers was diminished to some extent. The logistic regression models suggested an increasing risk of elevated γ-GT with smoking in particular in the higher alcohol consumption categories, no effect being evident in teetotalers. The effect estimates were somewhat attenuated by adjustment for covariables in addition to age and BMI. Similar patterns also appeared in the linear regression with *ln*(γ-GT) as the outcome: while smoking was not associated with changes in γ-GT in teetotalers, consuments of >90 g alcohol/day had a mean increase in γ-GT by about 200% if smoking heavily, but only by around 150% if never smoking.

In line with the tabulations (Table 3), the regression models of the BMI interaction showed a different picture (Supplementary Table S2). In particular, in both the more simple and the fully adjusted models, the odds of elevated γ-GT more clearly increased across rising smoking intensities in the normal and overweight category. A similar picture emerged in the models of *ln*-transformed γ-GT, which showed a monotonous dose-response relationship and statistically highly significant elevation of γ-GT by 23.2% in heavy (>20 cig./d) vs. never smokers in normal weight, but no clear association in obese subjects, in whom the confidence intervals around the estimated percent changes in the different smoking groups widely overlapped (difference in elevation in heavy vs. never smokers: 9.2%).

To summarize the above interactions in a more palatable way, we fitted smoking trend models allowing an interaction of smoking intensity with alcohol drinking groups or BMI. These estimated the increase in risk of elevated γ-GT and change in γ-GT concentrations associated with each additional 10 cigarettes per day. For the interaction with alcohol consumption—in line with the above results—an effect of smoking appeared only in strata other than teetotalers. In these models, the effect of smoking on γ-GT fairly consistently seemed to increase with higher alcohol co-consumption (Table 4). In the case of the interaction with BMI, the results showed a trend opposite to the original expectations: across models and adjustment sets, smoking intensity was associated with higher γ-GT in subjects with normal weight but not in obese subjects, with effect estimates being of intermediate magnitude in subjects with BMI 25 to <30 kg/m^2^ (Table 5). Statistical interaction tests were significant only for the fully adjusted models in case of the alcohol interaction, whereas the smoking × BMI interaction was significant throughout. When interactions with both alcohol and BMI were included in the same model, statistical support for either interaction diminished in particular in the fully adjusted logistic regression (*P*_alcohol_ = 0.066; *P*_BMI_ = 0.080), whereas it was more clearly preserved in the linear regression models with *ln*(γ-GT) as the outcome (*P*_alcohol_ = 0.021; *P*_BMI_ = 0.0022).

**Table 4:** Regression of γ-GT and smoking, by alcohol consumption category.

	Association of smoking (per additional 10 cig./d) with γ-GT concentrations within drinking strata															
Alcohol consumption	OR, 95% CI				Fully adj. OR, 95% CI				% change in γ-GT, 95% CI				Fully adj. % change in γ-GT, 95% CI			
None	0.95	0.80	-	1.13	0.94	0.79	-	1.12	0.38	-2.80	-	3.67	0.67	-2.47	-	3.90
Occasional	1.07	1.00	-	1.15	1.11	1.04	-	1.19	3.26	1.40	-	5.16	4.38	2.52	-	6.26
1-30 g/day	1.08	0.95	-	1.23	1.13	0.99	-	1.28	4.56	0.79	-	8.48	5.69	1.95	-	9.57
31-60 g/day	1.17	1.07	-	1.27	1.21	1.11	-	1.32	4.75	1.98	-	7.60	5.80	3.05	-	8.63
61-90 g/day	1.20	1.07	-	1.34	1.24	1.10	-	1.38	7.24	3.40	-	11.2	8.20	4.40	-	12.1
>90 g/day	1.23	1.10	-	1.37	1.27	1.13	-	1.42	6.19	2.44	-	10.1	7.74	4.01	-	11.6
Interaction^b^		*P* = 0.069				*P* = 0.039				*P* = 0.087				*P* = 0.028		

a All models adjusted for age, BMI, and alcohol consumption category; 'fully adjusted' models additionally adjusted for nationality, occupational group, prevalent diabetes, prevalent ischemic heart disease, prevalent hypertension.

b *P*-values refer to a 5 degrees of freedom Wald test for the significance of an interaction term of the smoking trend variable with the alcohol drinking category.

Results of trend models^a^ (median smoking intensity of 0, 10, 20, and 30 cigarettes/day assigned to individuals belonging to the different smoking intensity classes; former smokers excluded) estimating the effects of smoking on serum γ-GT levels nested in alcohol consumption intensity strata.

**Table 5:** Regression of γ-GT and smoking, by body mass index category.

	Association of smoking (per additional 10 cig./d) with γ-GT concentrations within BMI strata															
Body mass index	OR, 95% CI				Fully adj. OR, 95% CI				%change, 95% CI				Fully adj. %change, 95% CI			
<25 kg/m^2^	1.21	1.12	-	1.30	1.24	1.15	-	1.34	6.18	4.30	-	8.09	7.29	5.42	-	9.19
25 to <30 kg/m^2^	1.11	1.05	-	1.18	1.14	1.08	-	1.21	3.07	1.33	-	4.84	3.90	2.18	-	5.65
≥30 kg/m^2^	1.02	0.93	-	1.13	1.07	0.97	-	1.18	0.45	-2.54	-	3.54	1.89	-1.09	-	4.96
Interaction^b^		*P* = 0.022				*P* = 0.040				*P* = 0.0033				*P* = 0.0032		

a All models adjusted for age, alcohol consumption, and BMI category; 'fully adjusted' models additionally adjusted for nationality, occupational group, prevalent diabetes, prevalent ischemic heart disease, prevalent hypertension.

b *P*-values refer to a 2 degrees of freedom Wald test for the significance of an interaction term of the smoking trend variable with the BMI category.

Results of trend models^a^ estimating the effects of smoking on serum γ-GT levels nested in BMI strata.

To examine whether these interactions were due to residual covariance between alcohol consumption, BMI and smoking intensity within the drinking or BMI strata (e.g., among normal weight subjects, those with higher BMI could tend to have higher smoking intensity, potentially explaining a positive association of smoking with γ-GT in this stratum), we examined Spearman correlation coefficients between the continuous variables “cigarettes smoked per day” and “alcohol consumed per day” separately in each alcohol drinking stratum, and between “cigarettes smoked per day” and continuous BMI separately in each BMI stratum. For alcohol, all four correlation coefficients were positive and <0.09, and the coefficients for BMI ranged from −0.035 to 0.015, suggesting that such residual covariance was not responsible for the observations.

### Sensitivity Analyses

To check if our results were distorted by outlying values, we excluded subjects with γ-GT >246 U/L, consumption of >200 gram alcohol daily, smoking more than 60 cigarettes per day, or having a BMI of >36.5 kg/m^2^, i.e. those with values beyond the 99th percentile of the respective variable. This reduced the number of subjects contributing to the fully adjusted trend model from 12,696 (former smokers excluded) to 12,415 and mildly strengthened the observed effect modification by alcohol consumption with % changes per 10 additional cigarettes per day of 0.78, 4.02, 6.28, 4.81, 7.81, and 9.15 with increasing alcohol consumption intensities (interaction *P* = 0.0067). The interaction with BMI appeared somewhat attenuated, % changes per 10 cigarettes being 6.7, 3.9, and 2.2 in subjects with BMI <25, 25 to <30, and ≥30 kg/m^2^, respectively (interaction *P* = 0.012).

### Replication Study

Given the unexpected direction of the interaction of smoking with BMI, we attempted a direct replication of this finding using data from the independent ESTHER cohort, in which we had first described the smoking × alcohol interaction [13]. As shown in Table 6, the patterns of association emerging were very similar to the main analyses. Although the interaction term was statistically significant only in the fully adjusted linear regression model with *ln*(γ-GT) as the outcome and fitted to both sexes combined (i.e. the statistically most powerful model), smoking intensity consistently showed a stronger association with γ-GT in normal weight than in obese subjects, a possible exception being the logistic model in females.

**Table 6:** Replication analysis in the ESTHER study.

			γ-GT in U/L (at 25 °C)^b^		Association of smoking (per 10 cig./d) with γ-GT within BMI strata							
Model	BMI	n (elevated)	Geom. mean, 95% CI		Fully adjusted OR^b^, 95% CI				Fully adj. % change in γ-GT, 95% CI			
Whole population	All subjects	5354 (19%)	17.6	17.3–17.9								
	<25 kg/m^2^	1650 (14%)	15.2	14.7–15.7	1.21	1.06	-	1.38	9.14	5.88	-	12.5
	25 to <30 kg/m^2^	2362 (20%)	18.2	17.7–18.7	1.14	1.02	-	1.26	5.04	2.21	-	7.95
	≥30 kg/m^2^	1342 (24%)	20.0	19.4–20.7	1.02	0.89	-	1.15	1.59	-1.75	-	5.04
	Interaction^c^					*P* = 0.14				*P* = 0.0061		
Males only	All subjects	1768 (29%)	22.8	22.1–23.5								
	<25 kg/m^2^	472 (26%)	20.7	19.4–22.0	1.28	1.08	-	1.52	11.5	6.15	-	17.1
	25 to <30 kg/m^2^	904 (28%)	22.9	22.0–23.9	1.19	1.04	-	1.35	6.84	2.89	-	10.9
	≥30 kg/m^2^	392 (35%)	25.3	23.7–27.1	1.10	0.93	-	1.31	3.61	-1.57	-	9.06
	Interaction^c^					*P* = 0.47				*P* = 0.12		
Females only	All subjects	3586 (14%)	15.5	15.2–15.8								
	<25 kg/m^2^	1178 (9%)	13.4	13.0–13.9	1.07	0.86	-	1.33	6.87	2.68	-	11.2
	25 to <30 kg/m^2^	1458 (14%)	15.8	15.3–16.2	1.07	0.87	-	1.31	2.91	-1.41	-	7.42
	≥30 kg/m^2^	950 (19%)	18.2	17.5–18.9	0.93	0.75	-	1.14	0.35	-4.15	-	5.06
	Interaction^c^					*P* = 0.55				*P* = 0.11		

a Models adjusted for age (5-year groups), alcohol drinking (none, 1-39, 40-99, 100-199, ≥200 g/w), coffee consumption (none, <1, 1, >1 cup/d), sex, and BMI category.

b The assay used in the ESTHER study measured γ-GT at 37 °C, and the logistic models predicted elevated γ-GT defined according to the male cutoff (>50 U/L at 37 °C) provided by the manufacturer. Geometric means in the table refer to values at 25 °C, calculated by multiplying the 37 °C values with a conversion factor of 0.57 (Thomas L, et al. 2005; *J Lab Med* 29:301).

c *P*-values refer to a 2 degrees of freedom Wald test for the significance of an interaction term of the smoking trend variable with the BMI category.

Given are results of trend models^a^ (median smoking intensity of 0, 8, 20, 30 and 40 cigarettes/day assigned to individuals belonging to the different smoking intensity classes; former smokers excluded) estimating the effects of smoking on serum γ-GT levels nested in BMI strata.

### Interaction of Alcohol Consumption with BMI

As shown in Table 7, there also was a statistically significant interaction of alcohol consumption with BMI with respect to γ-GT. Alcohol consumption was positively associated with γ-GT in all BMI strata, but in particular the logistic regression model of the primary study population suggested a somewhat more pronounced effect in normal weight subjects. In the linear regression models with *ln*(γ-GT) as the outcome, the estimates were more similar in normal weight and obese subjects, and slightly lower in those being overweight.

**Table 7:** Interaction analysis of alcohol with BMI.

Study population	Body mass index	Association of alcohol (per additional 10 g/d) with γ-GT concentrations in each BMI stratum							
		Fully adjusted OR, 95% CI				Fully adj. %change, 95% CI			
Primary study	<25 kg/m^2^	1.33	1.29	-	1.37	11.0	10.2	-	11.8
	25 to <30 kg/m^2^	1.23	1.20	-	1.26	8.91	8.16	-	9.66
	≥30 kg/m^2^	1.24	1.19	-	1.29	9.42	8.18	-	10.7
	Interaction^b^		*P* = 0.0001				*P* = 0.0006		
ESTHER (whole study population)	<25 kg/m^2^	1.33	1.22	-	1.46	10.3	7.77	-	12.8
	25 to <30 kg/m^2^	1.24	1.16	-	1.32	7.40	5.58	-	9.25
	≥30 kg/m^2^	1.26	1.16	-	1.37	10.3	7.69	-	13.0
	Interaction^b^		*P* = 0.39				*P* = 0.081		

a Models are adjusted as described in Table 5 (primary study) and Table 6 (ESTHER study), but include smoking categories instead of the smoking intensity trend variable.

b *P*-values refer to a 2 degrees of freedom Wald test for the significance of an interaction term of the alcohol drinking variable with the BMI category.

Given are results of trend models^a^ estimating the association of alcohol consumption with serum γ-GT levels nested in BMI strata.

## Discussion

The findings in this large occupational cohort support the existence of opposite interactions between smoking and alcohol consumption, respectively smoking and BMI, with regard to elevations of serum γ-GT, a powerful marker of incident disease and mortality risk [22], [23]. The fact that smoking showed a more pronounced association with γ-GT in normal weight subjects, i.e. in the BMI stratum with low γ-GT, contrasts the situation in alcohol consumption and implies that the pathophysiological relationships linking these exposures are more complex than a mere accumulation of oxidative stress [13], [24], which both alcohol exposure and BMI had been assumed to reflect.

Previously studying the joint effects of cigarette smoking and alcohol consumption in the elderly general population-based ESTHER cohort in Germany, we had been able to ascribe statistical significance to a detrimental interaction between these two behaviours with respect to γ-GT [13]. The ESTHER cohort predominantly featured low to moderate consumption intensities, in particular with respect to drinking. Likewise, the very first study reporting on the interaction of interest—which was based on a socioeconomically somewhat more advantaged occupational cohort of mainly middle class (ABC1) subjects—was hampered by small numbers of subjects in joint high exposure categories [14], but consistent data have very recently been presented based on middle-aged British men from the general population [15]. In synthesis with the results cited above, the findings in the present report suggest that smoking and alcohol consumption aggravate each other's effect as determinants of serum γ-GT levels.

The associations of the individual exposures and covariables with serum γ-GT generally were in line with the pertinent literature [1], [3], [4], [9], [10], [11], [12] as well as our own analyses conducted on smaller subcohorts of the present study population enrolled during the early recruitment period [6], [8]. In case of the somewhat inconsistent prior evidence regarding smoking and γ-GT, the absence of a smoking main effect in teetotalers was in line with our previous work in ESTHER [13]. Despite the repeated description of a smoking × alcohol interaction, physiological mechanisms leading to the associations observed remain a matter of speculation. As suggested before, the cumulative oxidative stress from joint exposure to cigarette smoking and ethanol consumption might more easily exceed some threshold than would either exposure alone [11], [25], [26]. However, if this was the sole or main explanation, we would have expected the association between γ-GT and smoking to escalate also over increasing levels of BMI, likewise associated with higher oxidative stress [16]. In contrast, we observed an intriguingly opposite pattern of effect modification between smoking and BMI. This interaction apparently had not been examined in the published literature before, but its immediate replication in a second cohort strengthened our confidence in this finding. One might consider some form of dilution effect to play a role—larger body mass (and volume) in obesity possibly diluting cigarette exposure in some way—, but this similarly would be expected to apply to alcohol consumption as well. Whereas previous reports of alcohol consumption being associated with γ-GT elevation to a greater extent in subjects with higher rather than lower BMI render this hypothesis unlikely [11], [24], pertinent explorations in the present study were somewhat inconsistent and could not conclusively resolve this issue. Alternatively seeing the culprit in converging inflammatory stimuli [24], [27] likewise would not help to explain the opposite patterns for interactions of smoking with BMI and alcohol. Intriguingly, γ-GT has recently been suggested to be a marker of xenobiotics exposure in the general population [28]. As tobacco smoke contains a multitude of toxic substances that might be related to γ-GT in this way, the interaction pattern with BMI could be considered consistent with the hypothesis that such substances contained in cigarette smoke lose their relevance for γ-GT levels in obesity, where other xenobiotics bioaccumulated in adipose tissue might already predominate the determination of γ-GT.

As is common in self-report studies of harmful behaviours, participants might have tended to embellish their smoking and alcohol drinking habits [29], [30]. Due to our study setting, no biospecimen could be obtained for the purpose of validating exposure information. However, trend estimates as calculated for smoking within the drinking intensity strata are unlikely to be biased upwards by misclassification [31]. It also might appear reasonable to assume that misreporting of smoking status and intensity should occur very predominantly as underreporting, to a similar extent regardless of alcohol consumption intensity, and non-differential with respect to γ-GT. In the presence of an effect of smoking on γ-GT, false classification as ‘never smoking’ would raise the probability of elevated γ-GT in the reference category within each alcohol drinking group, most likely attenuating the effect estimates and diluting the statistical associations. It thus appears improbable that misclassification should be responsible for the observed interactions. A limitation of our study, however, remains the consideration of exposure at one point in time only. Future studies might try to account for cigarette pack-years and measures of life-time alcohol drinking rather than current consumption intensities.

A strength of the present study was the substantial sample size, which allowed precise estimation of associations over a wide range of exposure intensities and in detailed joint exposure categories. The restriction to male construction workers (with only a small comparison group of white collar workers) furthermore ensured an otherwise rather high homogeneity of the study population. The multiple regression methods applied to additionally control for important confounding variables further limit the potential for systematic error in the estimated associations described in the present report.

In conclusion, the confirmation of a detrimental interaction between smoking and alcohol consumption as determinants of serum γ-GT levels as described in this report supports the hypothesis that such an interaction exists and is of relevance not only at low to moderate consumption levels as frequently encountered in the general population [13], but also in higher exposure groups as observed as part of our special study cohort. However, the novel finding of an unexpectedly opposite interaction between smoking and BMI, which had been examined in order to confirm the hypothesis of excess oxidative stress being responsible for the smoking × alcohol interaction, highlights the surprisingly limited understanding of the underlying pathophysiological relationships. The virtual absence of definite knowledge pertaining to an absolutely routine laboratory parameter and its interaction with some of the most classical epidemiological exposures should strongly stipulate additional research efforts in this area, preferably encompassing not only large-scale epidemiological, but also biomolecular approaches on the cellular level. Regardless of these future efforts, the apparent confirmation of a detrimental interaction of smoking and alcohol consumption with respect to γ-GT, an important predictor of morbidity, mortality, and disability [32], should motivate occupational health practitioners to rigorously address tobacco/alcohol co-use in their clientele, who depend on their physical integrity even more than the general population.

## Supporting Information

Table S1 Age- and body mass index-adjusted associations between smoking x alcohol consumption intensity strata and serum γ-GT levels. Reported are odds ratios (OR) from logistic regression models predicting γ-GT >28 U/L (at 25°C) and results from regression models predicting logarithmically transformed γ-GT (expressed as % change in concentration).
(0.11 MB DOC)

Table S2 Age- and alcohol consumption-adjusted associations between smoking x BMI strata and serum γ-GT levels. Reported are odds ratios (OR) from logistic regression models predicting γ-GT >28 U/L (at 25°C) and results from regression models predicting logarithmically transformed γ-GT (expressed as % change in concentration).
(0.07 MB DOC)
