# Supplementary material for: Smoking and γ-Glutamyltransferase: Opposite Interactions with Alcohol Consumption and Body Mass Index
Source: PLoS One. 2010 Sep 30;5(9):e13116. doi: 10.1371/journal.pone.0013116 (PMC2948041; doi:10.1371/journal.pone.0013116)
Supplement: Table S1 — Age- and body mass index-adjusted associations between smoking x alcohol consumption intensity strata and serum γ-GT levels. Reported are odds ratios (OR) from logistic regression models predicting γ-GT >28 U/L (at 25°C) and results from regression models predicting logarithmically transformed γ-GT (expressed as % change in concentration). (0.11 MB DOC) [file pone.0013116.s001.doc]

| **Supplementary Table S1.** | | | | | | | | | | | | | | | | | | | |
| --- | --- | --- | --- | --- | --- | --- | --- | --- | --- | --- | --- | --- | --- | --- | --- | --- | --- | --- | --- |
| Alcohol consumption |  |  | OR for elevated γ-GT, 95% CI | | | | | | | |  | % change in γ-GT, 95% CI | | | | | | | |
|  | Smoking |  | Adjusted | | | | Fully adjusted* | | | |  | Adjusted | | | | Fully adjusted* | | | |
|  |  |  |  |  |  |  |  |  |  |  |  |  |  |  |  |  |  |  |  |
| None | Never |  | 1 | ref. |  |  | 1 | ref. |  |  |  | 0 | ref. |  |  | 0 | ref. |  |  |
|  | <20 cig./d |  | 1.34 | 0.83 | - | 2.16 | 1.38 | 0.84 | - | 2.26 |  | 1.13 | -8.28 | - | 11.5 | 3.71 | -5.77 | - | 14.1 |
|  | 20 cig./d |  | 0.69 | 0.42 | - | 1.15 | 0.71 | 0.42 | - | 1.19 |  | -0.67 | -8.92 | - | 8.33 | 1.76 | -6.57 | - | 10.8 |
|  | >20 cig./d |  | 1.10 | 0.62 | - | 1.97 | 0.98 | 0.54 | - | 1.79 |  | 2.95 | -8.46 | - | 15.8 | 1.17 | -9.85 | - | 13.5 |
|  | Formerly |  | 0.88 | 0.53 | - | 1.47 | 0.88 | 0.52 | - | 1.47 |  | 2.30 | -7.54 | - | 13.2 | 2.89 | -6.82 | - | 13.6 |
|  |  |  |  |  |  |  |  |  |  |  |  |  |  |  |  |  |  |  |  |
| Occasional | Never |  | 2.35 | 1.77 | - | 3.13 | 1.83 | 1.37 | - | 2.44 |  | 22.7 | 15.6 | - | 30.2 | 13.9 | 7.35 | - | 20.9 |
|  | <20 cig./d |  | 2.79 | 2.07 | - | 3.77 | 2.28 | 1.68 | - | 3.10 |  | 29.0 | 21.0 | - | 37.4 | 21.8 | 14.3 | - | 29.7 |
|  | 20 cig./d |  | 2.62 | 1.95 | - | 3.52 | 2.20 | 1.63 | - | 2.97 |  | 30.0 | 22.2 | - | 38.4 | 23.8 | 16.4 | - | 31.7 |
|  | >20 cig./d |  | 3.06 | 2.21 | - | 4.23 | 2.59 | 1.86 | - | 3.60 |  | 36.7 | 26.9 | - | 47.2 | 30.0 | 20.8 | - | 39.9 |
|  | Formerly |  | 2.99 | 2.23 | - | 4.02 | 2.31 | 1.71 | - | 3.12 |  | 34.2 | 25.6 | - | 43.3 | 24.2 | 16.3 | - | 32.6 |
|  |  |  |  |  |  |  |  |  |  |  |  |  |  |  |  |  |  |  |  |
| 1-30 g/day | Never |  | 2.90 | 2.05 | - | 4.09 | 2.25 | 1.58 | - | 3.19 |  | 29.8 | 19.6 | - | 40.9 | 20.5 | 11.1 | - | 30.7 |
|  | <20 cig./d |  | 3.34 | 2.30 | - | 4.85 | 2.88 | 1.97 | - | 4.20 |  | 37.9 | 26.2 | - | 50.7 | 32.2 | 21.1 | - | 44.3 |
|  | 20 cig./d |  | 3.98 | 2.79 | - | 5.70 | 3.52 | 2.45 | - | 5.07 |  | 51.3 | 38.7 | - | 65.0 | 45.8 | 33.8 | - | 58.9 |
|  | >20 cig./d |  | 3.18 | 1.97 | - | 5.14 | 2.61 | 1.61 | - | 4.25 |  | 39.1 | 23.0 | - | 57.3 | 30.8 | 15.9 | - | 47.7 |
|  | Formerly |  | 2.89 | 1.98 | - | 4.23 | 2.19 | 1.49 | - | 3.23 |  | 39.1 | 26.4 | - | 53.1 | 27.6 | 16.1 | - | 40.3 |
|  |  |  |  |  |  |  |  |  |  |  |  |  |  |  |  |  |  |  |  |
| 31-60 g/day | Never |  | 4.52 | 3.32 | - | 6.15 | 3.50 | 2.56 | - | 4.80 |  | 62.5 | 50.9 | - | 75.0 | 49.7 | 39.1 | - | 61.2 |
|  | <20 cig./d |  | 5.80 | 4.25 | - | 7.91 | 4.84 | 3.53 | - | 6.65 |  | 74.4 | 61.8 | - | 87.9 | 64.5 | 52.7 | - | 77.1 |
|  | 20 cig./d |  | 7.01 | 5.20 | - | 9.46 | 5.91 | 4.36 | - | 8.03 |  | 81.6 | 69.2 | - | 94.8 | 71.8 | 60.2 | - | 84.2 |
|  | >20 cig./d |  | 6.94 | 4.97 | - | 9.70 | 5.86 | 4.17 | - | 8.25 |  | 86.6 | 71.2 | - | 103.3 | 76.2 | 61.8 | - | 91.8 |
|  | Formerly |  | 6.88 | 5.07 | - | 9.34 | 5.35 | 3.91 | - | 7.31 |  | 83.0 | 69.7 | - | 97.4 | 68.1 | 55.9 | - | 81.2 |
|  |  |  |  |  |  |  |  |  |  |  |  |  |  |  |  |  |  |  |  |
| 61-90 g/day | Never |  | 7.27 | 5.14 | - | 10.3 | 5.72 | 4.01 | - | 8.15 |  | 92.9 | 75.6 | - | 111.8 | 77.5 | 61.8 | - | 94.8 |
|  | <20 cig./d |  | 9.67 | 6.71 | - | 13.9 | 7.54 | 5.18 | - | 11.0 |  | 125.8 | 104.2 | - | 149.7 | 106.9 | 87.3 | - | 128.5 |
|  | 20 cig./d |  | 13.6 | 9.80 | - | 18.8 | 11.5 | 8.28 | - | 16.1 |  | 138.7 | 119.5 | - | 159.5 | 124.4 | 106.5 | - | 143.9 |
|  | >20 cig./d |  | 11.4 | 7.94 | - | 16.5 | 9.50 | 6.55 | - | 13.8 |  | 136.5 | 114.0 | - | 161.4 | 121.1 | 100.3 | - | 144.1 |
|  | Formerly |  | 11.6 | 8.12 | - | 16.6 | 8.37 | 5.81 | - | 12.1 |  | 143.4 | 120.7 | - | 168.4 | 115.2 | 95.3 | - | 137.1 |
|  |  |  |  |  |  |  |  |  |  |  |  |  |  |  |  |  |  |  |  |
| >90 g/day | Never |  | 13.2 | 9.02 | - | 19.2 | 9.73 | 6.62 | - | 14.3 |  | 182.8 | 154.6 | - | 214.0 | 151.6 | 126.9 | - | 179.1 |
|  | <20 cig./d |  | 15.8 | 10.8 | - | 22.9 | 12.6 | 8.58 | - | 18.5 |  | 194.4 | 165.5 | - | 226.5 | 169.0 | 142.8 | - | 197.9 |
|  | 20 cig./d |  | 18.2 | 13.2 | - | 25.3 | 15.1 | 10.8 | - | 21.1 |  | 197.2 | 173.4 | - | 223.0 | 176.4 | 154.4 | - | 200.3 |
|  | >20 cig./d |  | 24.9 | 17.7 | - | 34.9 | 20.3 | 14.4 | - | 28.7 |  | 242.0 | 213.8 | - | 272.6 | 216.5 | 190.7 | - | 244.7 |
|  | Formerly |  | 16.7 | 11.7 | - | 23.9 | 12.6 | 8.71 | - | 18.2 |  | 190.1 | 163.6 | - | 219.3 | 158.3 | 134.9 | - | 184.0 |
| * Adjusted for all variables shown in Table 1. | | | | |  |  |  |  |  |  |  |  |  |  |  |  |  |  |  |
