# Supplementary material for: Smoking and γ-Glutamyltransferase: Opposite Interactions with Alcohol Consumption and Body Mass Index
Source: PLoS One. 2010 Sep 30;5(9):e13116. doi: 10.1371/journal.pone.0013116 (PMC2948041; doi:10.1371/journal.pone.0013116)
Supplement: Table S2 — Age- and alcohol consumption-adjusted associations between smoking x BMI strata and serum γ-GT levels. Reported are odds ratios (OR) from logistic regression models predicting γ-GT >28 U/L (at 25°C) and results from regression models predicting logarithmically transformed γ-GT (expressed as % change in concentration). (0.07 MB DOC) [file pone.0013116.s002.doc]

| **Supplementary Table S2.** | | | | | | | | | | | | | | | | | | |
| --- | --- | --- | --- | --- | --- | --- | --- | --- | --- | --- | --- | --- | --- | --- | --- | --- | --- | --- |
|  |  |  |  |  |  |  |  |  |  |  |  |  |  |  |  |  |  |  |
|  |  |  |  |  |  |  |  |  |  |  |  |  |  |  |  |  |  |  |
| Body mass index |  | OR for elevated γ-GT, 95% CI | | | | | | | |  | % change of γ-GT, 95% CI | | | | | | | |
|  | Smoking | Adjusted | | | | Fully adjusted* | | | |  | Adjusted | | | | Fully adjusted* | | | |
|  |  |  |  |  |  |  |  |  |  |  |  |  |  |  |  |  |  |  |
|  |  |  |  |  |  |  |  |  |  |  |  |  |  |  |  |  |  |  |
| <25 kg/m² | Never | 1 | ref. |  |  | 1 | ref. |  |  |  | 0 | ref. |  |  | 0 | ref. |  |  |
|  | <20 cig./d | 1.20 | 0.95 | - | 1.51 | 1.28 | 1.01 | - | 1.62 |  | 4.16 | -1.01 | - | 9.60 | 6.46 | 1.27 | - | 11.9 |
|  | 20 cig./d | 1.46 | 1.18 | - | 1.80 | 1.62 | 1.30 | - | 2.00 |  | 10.8 | 5.64 | - | 16.3 | 14.7 | 9.42 | - | 20.3 |
|  | >20 cig./d | 1.74 | 1.38 | - | 2.21 | 1.87 | 1.48 | - | 2.38 |  | 20.5 | 13.5 | - | 27.8 | 23.2 | 16.2 | - | 30.6 |
|  | Formerly | 1.30 | 0.99 | - | 1.71 | 1.33 | 1.01 | - | 1.75 |  | 8.74 | 1.90 | - | 16.0 | 9.37 | 2.62 | - | 16.6 |
|  |  |  |  |  |  |  |  |  |  |  |  |  |  |  |  |  |  |  |
| 25 to <30 | Never | 2.27 | 1.85 | - | 2.78 | 2.16 | 1.76 | - | 2.65 |  | 32.0 | 25.9 | - | 38.4 | 29.1 | 23.2 | - | 35.3 |
| kg/m² | <20 cig./d | 2.81 | 2.26 | - | 3.50 | 2.81 | 2.26 | - | 3.51 |  | 41.9 | 34.4 | - | 49.9 | 41.3 | 33.9 | - | 49.1 |
|  | 20 cig./d | 2.98 | 2.42 | - | 3.67 | 3.09 | 2.50 | - | 3.81 |  | 41.5 | 34.5 | - | 48.8 | 42.0 | 35.0 | - | 49.3 |
|  | >20 cig./d | 3.07 | 2.43 | - | 3.87 | 3.08 | 2.43 | - | 3.90 |  | 45.1 | 36.5 | - | 54.2 | 43.6 | 35.3 | - | 52.5 |
|  | Formerly | 2.89 | 2.34 | - | 3.56 | 2.70 | 2.18 | - | 3.34 |  | 43.4 | 36.2 | - | 51.1 | 38.8 | 31.9 | - | 46.1 |
|  |  |  |  |  |  |  |  |  |  |  |  |  |  |  |  |  |  |  |
| ≥30 kg/m² | Never | 4.39 | 3.44 | - | 5.62 | 3.75 | 2.92 | - | 4.83 |  | 68.1 | 57.3 | - | 79.6 | 55.6 | 45.8 | - | 66.2 |
|  | <20 cig./d | 5.27 | 3.96 | - | 7.01 | 4.78 | 3.57 | - | 6.40 |  | 79.4 | 65.3 | - | 94.7 | 69.4 | 56.3 | - | 83.7 |
|  | 20 cig./d | 4.68 | 3.53 | - | 6.21 | 4.18 | 3.13 | - | 5.58 |  | 73.0 | 59.8 | - | 87.4 | 62.5 | 50.2 | - | 75.9 |
|  | >20 cig./d | 4.79 | 3.51 | - | 6.54 | 4.64 | 3.37 | - | 6.38 |  | 70.1 | 55.3 | - | 86.3 | 64.8 | 50.7 | - | 80.2 |
|  | Formerly | 5.70 | 4.43 | - | 7.31 | 4.67 | 3.61 | - | 6.04 |  | 85.1 | 72.6 | - | 98.4 | 68.6 | 57.4 | - | 80.6 |
|  |  |  |  |  |  |  |  |  |  |  |  |  |  |  |  |  |  |  |
| * Adjusted for all variables shown in Table 1. | | | | | | | | | | | | | | | | | | |
